# *Vibrio alginolyticus*: an emerging marine pathogen at the intersection of aquaculture, public health, and antimicrobial resistance

**DOI:** 10.3389/fvets.2026.1965608

**Published:** 2026-09-29

**Authors:** Songlin Wei, Jingmin Lin, Ruichao Xue, Wei Xu

**Affiliations:** 1 School of Information Engineering, Xiamen Ocean Vocational College, Xiamen, China; 2 Fujian Provincial Key Laboratory of Marine Physical and Geological Processes, Third Institute of Oceanography, Ministry of Natural Resources, Xiamen, China; 3 Department of Artificial Intelligence, Xiamen City University, Xiamen, China; 4 School of Basic Medical Sciences, Yichun University, Yichun, China; 5 Center for Research and Development, Aquabrain Biotech (Xiamen) Co., Ltd, Xiamen, China

**Keywords:** control strategies, environmental distribution, genomics, host-pathogen interactions, immune response, virulence mechanisms

## Abstract

*Vibrio alginolyticus* is a halophilic, Gram-negative marine bacterium that has emerged as a significant opportunistic pathogen affecting both marine animals and humans. As one of the most abundant vibrios in coastal and estuarine environments worldwide, *V. alginolyticus* causes substantial economic losses in aquaculture through vibriosis in fish, shrimp, and shellfish, while also being responsible for wound infections, otitis externa, and occasionally gastroenteritis in humans following seawater exposure or seafood consumption. The pathogenesis of *V. alginolyticus* is multifactorial, involving an arsenal of virulence determinants including secretion systems (type III, type VI, and type II), haemolysins, adhesins, biofilm formation, quorum sensing-regulated factors, and the recently recognized contributions of non-coding RNAs and outer membrane vesicles. Recent genomic analyses have revealed considerable genetic diversity among isolates and identified a ubiquitous presence of β-lactam resistance genes, raising concerns about natural resistance and the potential for horizontal gene transfer. The interplay between rising seawater temperatures, intensive aquaculture practices, and increasing antimicrobial resistance has positioned *V. alginolyticus* as an emerging pathogen of growing concern. This Review synthesizes current knowledge on the epidemiology, genomics, virulence mechanisms, host interactions, and control strategies of *V. alginolyticus*, with an emphasis on the molecular determinants of pathogenesis, including the regulatory networks governing adhesion, biofilm formation, and metabolic adaptation, and the challenges posed by antimicrobial resistance. We also discuss promising alternative control approaches, including phage therapy, vaccination, and probiotic strategies, and identify key knowledge gaps that warrant future investigation.

## 1 Introduction

The genus *Vibrio* encompasses a diverse group of Gram-negative, facultatively anaerobic bacteria that are ubiquitous in aquatic environments. Among the more than 100 recognized *Vibrio* species, a subset has gained prominence as human and animal pathogens, including *Vibrio cholerae, Vibrio parahaemolyticus*, and *Vibrio vulnificus* (1). *Vibrio alginolyticus*, historically classified as biotype 2 of *V. parahaemolyticus*, has long been considered primarily an environmental organism with modest pathogenic potential. This perception is changing.

*V. alginolyticus* is now recognized as one of the most common *Vibrio* species in seawater and seafood, and its association with disease outbreaks in both aquaculture settings and human clinical cases has intensified over the past two decades (2). In aquaculture, *V. alginolyticus* causes vibriosis in a wide range of commercially important species, including gilthead seabream (*Sparus aurata*), orange-spotted grouper (*Epinephelus coioides*), Pacific white shrimp (*Penaeus vannamei*), and kuruma prawn (*Penaeus japonicus*). The disease manifests as haemorrhagic septicaemia, skin ulcerations, and high mortality rates, leading to substantial economic losses (3). In humans, *V. alginolyticus* is most frequently associated with wound infections and otitis externa following exposure to marine environments, though cases of gastroenteritis and, rarely, septicaemia have also been documented (1, 4).

Several converging factors have contributed to the rising prominence of *V. alginolyticus* as a pathogen. Climate change has led to warming of coastal waters, expanding the geographic range and seasonal window of *Vibrio* proliferation (5). The expansion and intensification of aquaculture have created densely populated rearing conditions that facilitate pathogen transmission (6). Meanwhile, the widespread use of antibiotics in aquaculture has selected for resistant strains, complicating treatment options (7). These developments underscore the need for a comprehensive understanding of *V. alginolyticus* biology, pathogenesis, and epidemiology to inform effective surveillance and control strategies.

This Review provides a synthesis of the current state of knowledge on *V. alginolyticus*. We begin by examining its environmental distribution, ecological niches, and epidemiological patterns. We then discuss genomic features and population structure, followed by a detailed analysis of the molecular mechanisms underlying its pathogenicity, including the hierarchical regulatory networks that govern adhesion, flagellar assembly, chemotaxis, and metabolic adaptation. The host immune response, detection methods, and control strategies are subsequently addressed. We also highlight the emerging roles of non-coding RNAs and outer membrane vesicles in virulence regulation. We conclude by identifying priority areas for future research.

## 2 Environmental distribution and epidemiology

### 2.1 Ecological niche and environmental drivers

*V. alginolyticus* is an autochthonous member of marine and estuarine microbial communities, distributed globally in temperate, subtropical, and tropical coastal waters. The bacterium exhibits a marked preference for warm, moderately saline environments, with its abundance strongly correlated with water temperature and salinity (8). Seasonal patterns of *V. alginolyticus* prevalence are well documented, with peak concentrations occurring during warmer months when water temperatures exceed 15–20 °C. Detection rates in environmental samples vary considerably by region: studies in Asia have reported *V. alginolyticus* in more than 80% of water samples, whereas detection rates in US and European shellfish at harvest range between 5 and 50% (9).

The ecological success of *V. alginolyticus* can be attributed to several physiological adaptations. As a halophilic organism, it requires sodium chloride for growth and can tolerate salinities ranging from brackish to fully marine conditions. It forms large, yellow (sucrose-fermenting) colonies on thiosulfate-citrate-bile salts-sucrose (TCBS) agar and exhibits pronounced swarming on non-selective solid media (10). The bacterium is capable of both planktonic and biofilm-associated lifestyles, with the latter providing enhanced survival under environmental stress (11).

Integrated surveillance in mariculture-impacted coastal ecosystems, such as Dongshan Bay in southern China, has provided critical insights into the seasonal dynamics of *Vibrio* populations. Annual *Vibrio* abundance in such semi-enclosed bays ranges from 20 to 11,600 CFU mL^−1^, with temperature identified as the most significant positive correlate (12). Community composition shifts markedly across seasons: *V. campbellii* dominates in summer, *V. fortis* in autumn, and species richness peaks in spring, while pathogenic vibrios are rarely isolated in winter (12, 13). These findings underscore the pronounced seasonality of *Vibrio* abundance and composition, with important implications for aquaculture disease forecasting.

### 2.2 Aquaculture impact

The economic impact of *V. alginolyticus* on aquaculture is substantial and growing. Disease outbreaks in Mediterranean aquaculture have become increasingly frequent, driven by the semi-enclosed nature of the Mediterranean Sea, rising seawater temperatures, and intensive farming practices (3). Affected species include finfish (gilthead seabream, European seabass, groupers), crustaceans (shrimp, prawns), and bivalve molluscs (oysters, clams). In Pacific oyster (*Crassostrea gigas*) larvae, *V. alginolyticus* strain CG-1 induced dose-dependent mortality with an LD50 of 5.04 × 106 CFU/mL, with clinical symptoms comparable to those caused by the well-known oyster pathogen *V. coralliilyticus* (14). The global expansion of aquaculture production, now supplying nearly half of all fish consumed worldwide, has created favorable conditions for the dissemination of *V. alginolyticus* and other *Vibrio* pathogens (6).

### 2.3 Transmission and human disease

Human infections with *V. alginolyticus* are overwhelmingly associated with exposure to seawater or consumption of contaminated seafood. Wound infections typically occur when an individual with an open cut or abrasion wades or swims in coastal waters harboring the bacterium. The resulting cellulitis is generally mild, characterized by localized inflammation and seropurulent exudate, though more severe infections can occur in immunocompromised individuals. Otitis externa, or swimmer's ear, is another common presentation, particularly among individuals with prolonged water exposure. Gastroenteritis following ingestion of raw or undercooked seafood has been reported but is less common than infections caused by *V. parahaemolyticus* (4, 15). Immunocompromised patients and those with underlying chronic conditions are at increased risk of more severe outcomes, including bacteraemia (16).

## 3 Genomics diversity and population structure

A comprehensive comparative genomic analysis of 184 *V. alginolyticus* isolates has revealed substantial genetic diversity and a complex population structure (17). The population can be divided into three major lineages (L1, L2, and L3), each of which can be further subdivided into sublineages. Accessory genes, those not shared by all members of the species, are nonuniformly distributed among isolates and contribute to genomic differentiation. Importantly, the spread of certain post-evolutionary isolates (predominantly L3 strains from Chinese territorial seas) appears to be closely linked to human activities, whereas more ancestral strains (L1 and L2) tend to be locally endemic and form clonal complex groups (17).

This population structure has implications for both pathogenesis and epidemiology. The uneven distribution of accessory genes, many of which are located on mobile genetic elements, facilitates the acquisition of virulence determinants and antibiotic resistance genes (18). A study of 55 *V. alginolyticus* isolates from seafood and freshwater products in China identified 34 distinct sequence types (STs) by multilocus sequence typing, with ST421, ST166, ST523, ST516, and ST507 being the most prevalent (19). This considerable genetic diversity underscores the adaptive capacity of *V. alginolyticus* and the challenges it poses for surveillance and control.

## 4 Virulence mechanisms

### 4.1 Virulence-associated genes

The genome of *V. alginolyticus* encodes a diverse array of virulence-associated genes (20). These include components of the type III secretion system (T3SS), extracellular enzymes, motility and chemotaxis regulators, outer membrane proteins, iron acquisition systems, and antibiotic resistance genes. A genome-wide analysis of potential virulence factors identified genes associated with toxin production, adherence, motility, chemotaxis, and the T3SS as the most prominent (21). The presence of these genes varies among isolates, contributing to differences in pathogenic potential.

### 4.2 Secretion systems

Secretion systems are central to the virulence of *V. alginolyticus*, enabling the delivery of effector proteins into host cells or the extracellular environment (Figure 1). The bacterium possesses at least three distinct secretion systems: type III secretion system (T3SS), type VI secretion system (T6SS), and type II secretion system (T2SS) (22).

**Figure 1:**
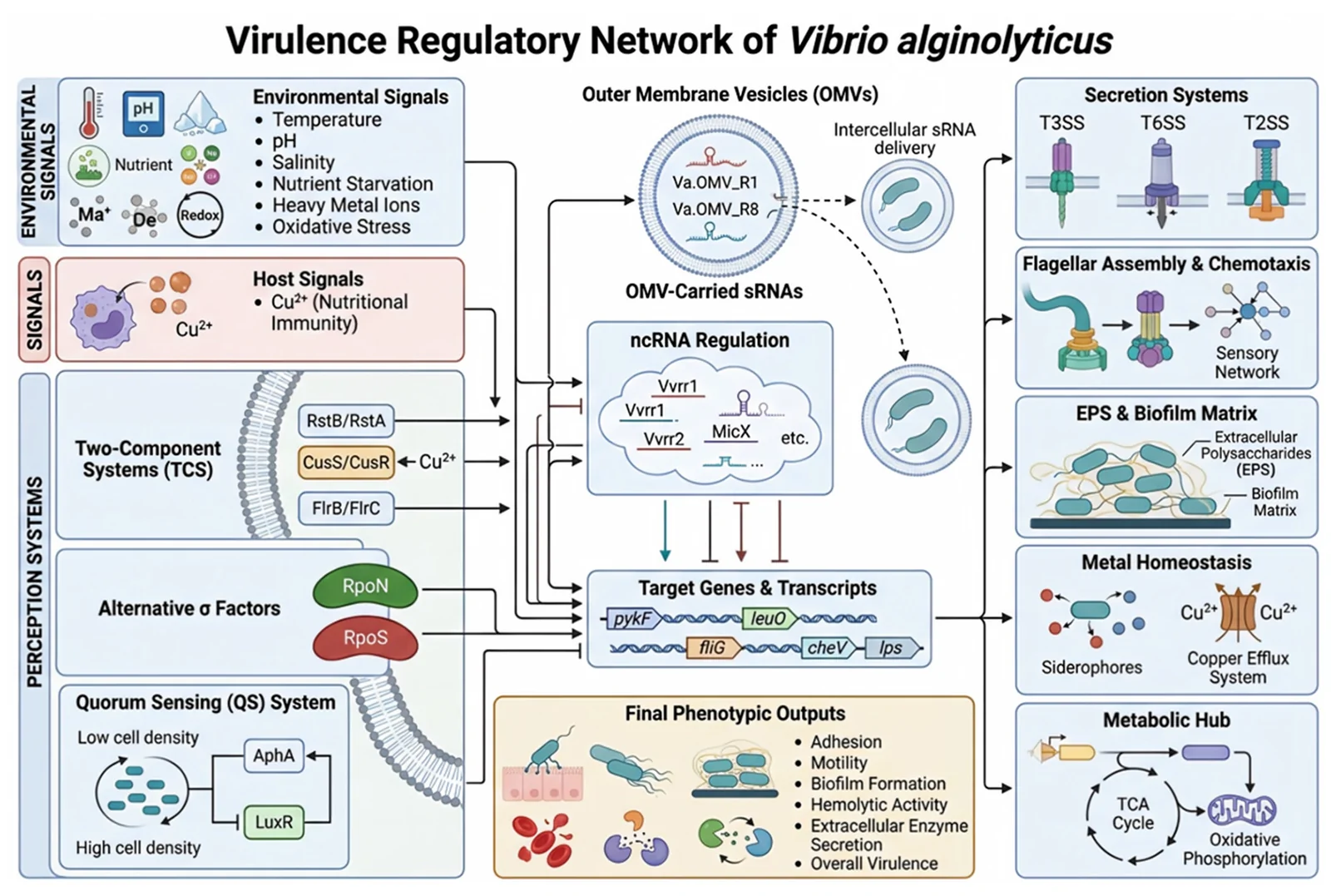
Hierarchical regulatory network governing virulence in *Vibrio alginolyticus*. This schematic illustrates the multilayered regulatory architecture integrating environmental and host-derived signals to control virulence outputs in *V. alginolyticus*. Environmental cues (temperature, pH, salinity, starvation, heavy metals and oxidative stress) and host-derived signals (copper ions from nutritional immunity) are sensed by membrane-bound two-component systems (RstB/RstA, CusS/CusR, FlrB/FlrC), alternative sigma factors (RpoN, RpoS), and the quorum-sensing circuitry (AphA/LuxR). These signals converge on central regulatory hubs, including non-coding RNAs (Vvrr1, Vvrr2, MicX) and OMV-associated sRNAs (Va.OMV_R1/R8), which post-transcriptionally modulate target genes such as *pykF, leuO, fliG, cheV* and *lps*. Downstream effector pathways comprise type III, type VI and type II secretion systems, flagellar assembly and chemotaxis, exopolysaccharide (VPS) biosynthesis and biofilm matrix production, siderophore-mediated iron uptake and copper efflux, as well as metabolic hubs (TCA cycle and oxidative phosphorylation). The integrated output shapes key virulence phenotypes including adhesion, motility, biofilm formation, haemolytic activity, extracellular enzyme secretion and overall pathogenicity. Arrows indicate activation, whereas T-bars denote repression. Dashed arrows represent OMV-mediated intercellular delivery of sRNAs.

The T3SS is an essential virulence determinant. *V. alginolyticus* encodes a T3SS that translocates effector proteins directly into host cells, subverting host signaling pathways and promoting cell death. To date, four T3SS effectors have been identified in *V. alginolyticus*: VopQ, VopR, VopS, and VPA0450 (23). VopR, a 317-amino-acid protein encoded by a 954-bp gene, binds directly to phosphatidic acid, PtdIns (3,5)P_2_, and PtdIns (4,5)P_2_ on the host cell membrane. This binding activates the host ubiquitination system and induces apoptosis through the MKK (mitogen-activated protein kinase kinase) signaling pathway (24). VopS functions as a metabolic disruptor that promotes host cell ferroptosis by enhancing the synthesis of adrenic acid, depleting cellular glutathione, and increasing the accumulation of ferrous iron (25). The deletion of *vopS* reduces the ability of *V. alginolyticus* to secrete extracellular proteases and attenuates virulence (26).

The T6SS is a widespread protein secretion apparatus used by Gram-negative bacteria to deliver toxic effector proteins into adjacent bacterial or host cells (27). *V. alginolyticus* possesses two complete T6SS gene clusters, designated T6SS1 and T6SS2 (28). Both systems are active at 30 °C, but only T6SS2 is also active at 37 °C, suggesting distinct roles in environmental versus host-associated contexts (29). The T6SS mediates interbacterial competition; *V. alginolyticus* can kill *Escherichia coli* in a T6SS-dependent manner (30). The haemolysin-coregulated protein Hcp2, a hallmark of T6SS2, has been implicated in motility, adhesive capacity, and pathogenicity (31). The T6SS is regulated by quorum sensing and the alternative sigma factor RpoN (32), and a complex regulatory connection exists between T6SS, quorum sensing, and c-di-GMP signaling through the phosphatase PppA encoded within the T6SS gene cluster (33, 34).

The T2SS also contributes to *V. alginolyticus* virulence. Components of the general secretory pathway, including SecA, SecD, SecF, YajC, and YidC, play critical roles in adhesion regulation. Gene silencing of *secA, secD, secF, yajC*, and *yidC* significantly reduces adhesion to fish mucus, with *secA*-RNAi and *yidC*-RNAi showing the most pronounced effects (35). The expression of these T2SS genes is sensitive to environmental factors including temperature, salinity, pH, and starvation, suggesting that the T2SS mediates adhesion regulation in response to environmental cues (35).

### 4.3 Haemolysins and toxins

Toxins constitute the most frequently identified category of virulence factors in *V. alginolyticus*, accounting for more than 50% of virulence-related keywords in bibliometric analyses (36). Haemolysins are the predominant toxins and function as pore-forming exotoxins that lyse erythrocytes and other mammalian cell membranes. *V. alginolyticus* produces three types of haemolysins: thermostable direct haemolysin (TDH), TDH-related haemolysin (TRH), and thermolabile haemolysin (TLH) (37). TLH is notable for its strong phospholipase activity rather than pore-forming activity, and it has been implicated in the induction of apoptosis through membrane damage (38). In addition to haemolysins, *V. alginolyticus* produces exfoliative toxins and microbial collagenase (39). The presence of *tdh* and *trh* genes, which are well-characterized virulence determinants in *V. parahaemolyticus*, has been documented in some *V. alginolyticus* strains (40). Surveillance studies in mariculture-impacted ecosystems have detected *tdh* in 6.32% and *trh* in 15.52% of atypical *Vibrio* isolates, indicating horizontal gene transfer of these virulence determinants across species boundaries (13) (Figure 1).

### 4.4 Adhesion, biofilm, and motility

Adhesion to host surfaces is a critical early step in infection, and *V. alginolyticus* employs multiple adhesins for this purpose. The toxin-coregulated pilus has been identified as a major motility-related virulence factor (41). Mannose-sensitive haemagglutinin type IV pili (MSHA) are also involved in adhesion and biofilm formation (42).

The hierarchical regulatory network governing adhesion involves multiple interconnected pathways. The flagellar assembly pathway, particularly genes encoding the filament (FliC), filament cap (FliD), L-ring (FlgH), and chaperone (FliS), plays an essential role in adhesion. Silencing of *fliC, fliD, flgH*, and *fliS* significantly impairs both motility and adhesion, though motility is not the sole mechanism through which the flagellar assembly pathway affects adhesion (43). The two-component regulatory system FlrA-FlrB-FlrC, which controls flagellar synthesis, directly regulates adhesion by modulating the expression of critical downstream genes including *fliA, flgH, fliS, fliD, cheR, cheV*, and the c-di-GMP-related gene *V12G01_22158*. Notably, silencing of *flrB* results in the most pronounced inhibition of flagellar assembly and adhesion, while silencing of *flrA* and *flrC* leads to upregulation of virulence genes including *toxT, ctxB*, and *hlyA*, suggesting that FlrA and FlrC repress certain virulence factors under normal conditions (44).

The chemotaxis pathway constitutes another critical regulatory layer. Genes encoding methyl-accepting chemotaxis proteins (MCPs), aerotaxis transducer (Aer), methylesterase (CheB), and methyltransferase (CheV) are essential for adhesion. Silencing of *mcp, aer, cheB*, and *cheV* not only reduces adhesion but also impairs chemotaxis, flagellar assembly, and motility. The expression of these genes is sensitive to temperature, pH, salinity, and starvation, indicating that environmental factors modulate adhesion through the chemotaxis pathway (45). Chemotaxis genes *cheA, cheB, cheR, cheV*, and *cheY* have also been shown to regulate adhesion in the related pathogen *V. harveyi* by affecting motility (46).

The RstB-RstA two-component system represents an additional regulatory node in the adhesion network. Silencing of *rstA* and *rstB* reduces adhesion, biofilm production, motility, haemolysis, and virulence, and their expression is responsive to temperature, pH, and starvation (47). Similarly, the general stress response sigma factor RpoS regulates multiple virulence phenotypes including adhesion, growth, haemolysis, biofilm production, motility, and virulence. RpoS modulates adhesion in response to temperature, pH, and starvation, but not salinity, suggesting pathway-specific environmental sensitivity (48).

Biofilm formation is a central feature of *V. alginolyticus* pathogenesis, providing protection against environmental stresses, antimicrobial agents, and host immune defenses (49). The biofilm matrix is composed primarily of extracellular polysaccharides, proteins, and nucleic acids. *Vibrio* exopolysaccharide (VPS) plays multiple roles, functioning both as a molecular glue for cell-cell and cell-surface adhesion and as a structural component of biofilms (50). The *vps* gene cluster, which is responsible for VPS production, is directly controlled by the alternative sigma factor RpoN. RpoN mediates biofilm formation by directly controlling the *vps* gene cluster and c-di-GMP synthetic metabolism (51). The stringent response regulators RelA and SpoT, which synthesize the alarmone (p)ppGpp, are involved in regulating cell motility, exopolysaccharide production, and biofilm formation (52, 53). The small non-coding RNA MicX has emerged as a critical regulator of biofilm formation; a Δ*micX* mutant exhibits reduced exopolysaccharide synthesis, biofilm formation, and alkaline serine protease production. Proteomic analysis of the Δ*micX* mutant identified 900 differentially expressed proteins, with downregulated proteins primarily engaged in biofilm formation, cellular communication, and transmembrane transport (54).

Motility, mediated by flagella, is another important virulence attribute. *V. alginolyticus* possesses a single polar flagellum that enables swimming motility in liquid environments and swarming over surfaces. The flagellum also contributes to adhesion and biofilm formation (55). Studies using real-time fluorescent imaging have revealed length-dependent flagellar growth in *V. alginolyticus* (56) (Figure 1).

### 4.5 Iron acquisition

Iron is an essential nutrient for bacterial growth, and *V. alginolyticus* has evolved multiple strategies to acquire iron from the host environment. The genome encodes iron acquisition systems, including siderophore-mediated uptake pathways (57). The biosynthetic gene clusters for the siderophore vibrioferrin exhibit genetic and structural variations across *Vibrio* species, including *V. alginolyticus* (58). These iron acquisition systems are critical for survival and proliferation within the host, where iron is sequestered by host proteins such as transferrin and lactoferrin.

The copper tolerance system CusS-CusR-CusCFBA exemplifies the intersection of metal homeostasis and virulence regulation. Copper stress, which arises from nutritional immunity during infection, activates the CusS-CusR two-component system, inducing expression of the *cusCFBA* efflux complex. Unexpectedly, activation of CusS-CusR by copper stress reduces adhesion, haemolysis, biofilm formation, motility, and overall virulence, despite conferring copper tolerance. This suggests that *V. alginolyticus* prioritizes copper homeostasis over virulence expression when facing metal stress, possibly as an energy-conservation strategy (59). Promoter region analysis, EMSA, and LacZ reporter assays confirmed that CusR directly regulates multiple virulence-related genes including *pilN, ompA, lptC, pykF, pflD*, and *feoA*, establishing a direct link between metal-sensing two-component systems and virulence gene regulation (59) (Figure 1).

## 5 Virulence regulatory mechanisms

Virulence in *V. alginolyticus* is coordinated by multilayered regulatory networks. Among these, quorum sensing and non-coding RNAs provide major cell-density-dependent and post-transcriptional regulatory mechanisms, respectively.

### 5.1 Quorum sensing

Quorum sensing (QS) is a cell-density-dependent regulatory mechanism that orchestrates many bacterial behaviors, including virulence and biofilm formation (60). In *V. alginolyticus*, QS tightly regulates pathogenicity. The QS circuit involves the production and detection of signaling molecules, predominantly N-acyl homoserine lactones (AHLs) (61). AphA, a master activator of the QS system at low cell density, can initiate the virulence cascade by positively regulating the expression of secretion systems, flagellar systems, and biofilm formation (62). At high cell density, LuxR becomes the dominant regulator. The small RNA MicX modulates QS by affecting the expression levels of LuxR and AphA (54) (Figure 1).

An intriguing and potentially clinically relevant finding is that QS positively regulates capsular polysaccharide (CPS)-dependent phage infection. QS upregulates the expression of the *ugd* gene, leading to increased synthesis of Autographiviridae phage receptor CPS. While this QS-mediated increase in phage susceptibility represents a trade-off, it also results in the upregulation of virulence factors, promoting biofilm development and enhanced capsular polysaccharide production. This observation has important implications for phage therapy, as inhibiting QS may reduce the therapeutic efficacy of CPS-specific phages (63).

### 5.2 Non-coding RNAs

Recent systematic screens have identified numerous non-coding RNAs (ncRNAs) that function as critical post-transcriptional regulators of virulence in *V. alginolyticus*. High-throughput sequencing combined with bioinformatics analysis revealed 32 ncRNAs commonly altered under adhesion-reducing stress conditions (Cu^2+^, Pb^2+^, Hg^2+^, and low pH) (64). Among these, seven ncRNAs, Candidate_103, Candidate_283, Candidate_424, Candidate_438, Candidate_442, Candidate_448, and Candidate_635, negatively regulate adhesion by targeting genes encoding type IV pilus assembly proteins (PilQ, PilM), chemotaxis proteins (MotB), and flagellar motor switch proteins (FliM) (65). Stable overexpression of these ncRNAs significantly reduced both target gene expression and adhesion ability, confirming their regulatory roles (65).

The ncRNA Vvrr1 (*Vibrio* virulence regulatory RNA 1) has been characterized as a key regulator of virulence. Overexpression of Vvrr1 reduces adhesion, biofilm formation, and virulence in *V. alginolyticus*. Proteomic analysis identified pyruvate kinase I (PykF) as a primary target of Vvrr1. GFP reporter gene analysis confirmed that Vvrr1 interacts with *pykF* mRNA, and silencing of *pykF* phenocopies the effects of Vvrr1 overexpression, reducing adhesion, biofilm formation, and virulence, while downregulating downstream genes including *ndk, eno, sdhB, glpF*, and *cysH* (66). The Vvrr1-PykF axis thus represents a critical regulatory node linking metabolism and virulence.

Furthermore, the ncRNA Vvrr1 has been shown to modulate the VarS/VarA-Csr system via the transcriptional regulator LeuO. Vvrr1 post-transcriptionally represses LeuO by targeting *leuO* mRNA and accelerating its decay. LeuO, in turn, directly represses *varS* transcription by binding to a conserved motif (ATTGGTGTTCAT) in the *varS* promoter. This repression alleviates inhibition of the Csr sRNAs (CsrB, CsrC, CsrD), which sequester the global regulator CsrA, leading to derepression of LuxO and subsequent modulation of exopolysaccharide biosynthesis and biofilm architecture (67). This multilayered regulatory circuit, from sRNA to transcription factor to two-component system to small RNA cascade, exemplifies the sophisticated integration of post-transcriptional and transcriptional control mechanisms in *V. alginolyticus* pathogenesis.

Building on this work, the ncRNA Vvrr2 has been shown to regulate biofilm formation through multiple target genes. Overexpression of Vvrr2 reduces adhesion, motility, biofilm formation, and virulence. Raman spectroscopy and confocal laser scanning microscopy revealed that Vvrr2 overexpression alters both the chemical composition and three-dimensional structure of biofilms, reducing extracellular polysaccharide, protein, and nucleic acid content (68). GFP reporter assays confirmed that Vvrr2 directly targets *fliG* (flagellar rotor protein), *cheV* (chemotaxis signal transduction protein), and *lps* (lipopolysaccharide synthesis), which are essential for motility, adhesion, and biofilm formation, respectively (68).

The oligopeptide permease (Opp) system, while not an ncRNA itself, functions within the broader regulatory network of adhesion. The *oppABCDF* gene cluster contributes to multiple steps of pathogenesis, including adhesion, biofilm production, and haemolytic activity. Silencing of *oppA, oppB, oppC, oppD*, and *oppF* reduces adhesion, peptide internalization, biofilm formation, haemolytic activity, and virulence, while expression of *oppABCDF* is sensitive to temperature, pH, and starvation (69) (Figure 1).

## 6 Outer membrane vesicles

Outer membrane vesicles (OMVs) have emerged as important mediators of *V. alginolyticus* pathogenesis and intercellular communication. *V. alginolyticus* produces OMVs with typical characteristics, spherical, membrane-bound structures ranging from 10 to 400 nm in diameter, containing the outer membrane protein OmpC (70). These OMVs carry a high abundance of small RNAs (sRNAs), which are protected from RNase degradation by the lipid membrane, indicating that OMVs serve as vehicles for sRNA delivery (70).

RNA sequencing of OMV-associated sRNAs identified 180 distinct sRNA sequences, with the ten most abundant sRNAs predicted to regulate genes involved in quorum sensing, secretion systems, two-component signal transduction, biofilm formation, and amino acid and carbohydrate metabolism (70). Two of these sRNAs, Va.OMV_R1 and Va.OMV_R8, were functionally characterized. Deletion of Va.OMV_R1 or Va.OMV_R8 reduced motility, extracellular matrix production, and biofilm formation by 52.7% and 58.7%, respectively. qRT-PCR validation confirmed that Va.OMV_R1 regulates target genes including *yscT, tkt1, aroB*, and *ygiP*, while Va.OMV_R8 regulates *clpV1, gcvA, mraY*, and *oppD* (70).

Crucially, OMVs from wild-type *V. alginolyticus* promote biofilm formation in a dose-dependent manner, accelerating biofilm development and increasing biomass. However, OMVs from Va.OMV_R1 or Va.OMV_R8 knockout strains exhibit diminished biofilm-promoting activity. GFP reporter assays and rifampicin chase experiments confirmed that Va.OMV_R1 promotes *yscT* expression while Va.OMV_R8 represses *gcvA* translation, establishing direct sRNA-mRNA interactions (70). These findings demonstrate that OMVs deliver functional sRNAs to recipient bacterial cells, modulating gene expression and biofilm formation through intercellular communication, a mechanism with significant implications for bacterial community behavior and pathogenesis (Figure 1).

The major virulence factors discussed above, including secretion systems, haemolysins, adhesins, motility apparatus, iron acquisition systems, regulatory circuits (quorum sensing, two-component systems, sigma factors, non-coding RNAs), and outer membrane vesicles, are summarized in Table 1 with their respective functions and key references.

**Table 1:** Major virulence factors of *Vibrio alginolyticus*.

Virulence factor category	Specific factors	Function
Secretion systems	T3SS (effectors VopQ, VopR, VopS, VPA0450)	Translocation of effectors into host cells; VopR induces apoptosis via MKK pathway (24); VopS induces ferroptosis (25)
	T6SS1 and T6SS2	Interbacterial competition; delivery of toxic effectors; T6SS2 active at 37 °C (28, 29)
	T2SS (SecA, SecD, SecF, YajC, YidC)	Secretion of extracellular enzymes; adhesion regulation (35)
Haemolysins and toxins	TDH, TRH, TLH	Pore-forming and phospholipase activities; cytolysis; apoptosis induction (37, 38)
	Exfoliative toxins, collagenase	Tissue damage (39)
Adhesion and biofilm	VPS (Vibrio exopolysaccharide)	Biofilm matrix component; cell-cell and cell-surface adhesion (50)
	MSHA pili	Adhesion; biofilm formation (42)
	Toxin-coregulated pilus	Motility-related virulence (41)
Motility	Polar flagellum	Swimming and swarming motility; adhesion (55)
Iron acquisition	Vibrioferrin siderophore	Iron acquisition from host (57, 58)
	CusS-CusR-CusCFBA	Copper tolerance; negatively regulates virulence under copper stress (59)
Regulation	Quorum sensing (AphA, LuxR)	Cell-density-dependent regulation of virulence; biofilm formation (60–62)
	sRNA MicX	Post-transcriptional regulation of biofilm and QS (54)
	RpoN	Regulation of *vps* cluster and biofilm formation (51)
	FlrA-FlrB-FlrC	Flagellar synthesis; adhesion regulation via downstream genes (44)
	RstB-RstA	Adhesion, biofilm, motility, haemolysis regulation (47)
	RpoS	Stress response; adhesion, growth, haemolysis, biofilm, motility, virulence (48)
	sRNA Vvrr1	Represses adhesion, biofilm, virulence via PykF and LeuO-VarS-Csr cascade (66, 67)
	sRNA Vvrr2	Represses biofilm formation via FliG, CheV, Lps (68)
Outer membrane vesicles	OMV-associated sRNAs (Va.OMV_R1, Va.OMV_R8)	Intercellular sRNA delivery; biofilm regulation (70)
Nutrient acquisition	OppABCDF	Oligopeptide transport; adhesion, biofilm, haemolysis, virulence (69)

T2SS, type II secretion system; T3SS, type III secretion system; T6SS, type VI secretion system; Vop, *Vibrio* outer protein; MKK, mitogen-activated protein kinase kinase; TDH, thermostable direct haemolysin; TRH, TDH-related haemolysin; TLH, thermolabile haemolysin; VPS, *Vibrio* exopolysaccharide; MSHA, mannose-sensitive hemagglutinin; QS, quorum sensing; sRNA, small regulatory RNA; OMV, outer membrane vesicle.

## 7 Host-pathogen interactions and immune response

The interaction between *V. alginolyticus* and its hosts involves complex molecular crosstalk that determines the outcome of infection (Figure 2). In aquatic organisms, the innate immune system provides the primary defense against *Vibrio* infections. Transcriptomic studies in bivalves have revealed that the response to *V. alginolyticus* infection involves coordinated regulation of pathogen recognition, oxidative stress, inflammatory signaling, apoptosis, and antimicrobial defense. In the clam *Mactra veneriformis*, infection triggers significant changes in multiple signaling pathways, including those involved in lysosomes, apoptosis, amino acid metabolism, and endocytosis (71).

**Figure 2:**
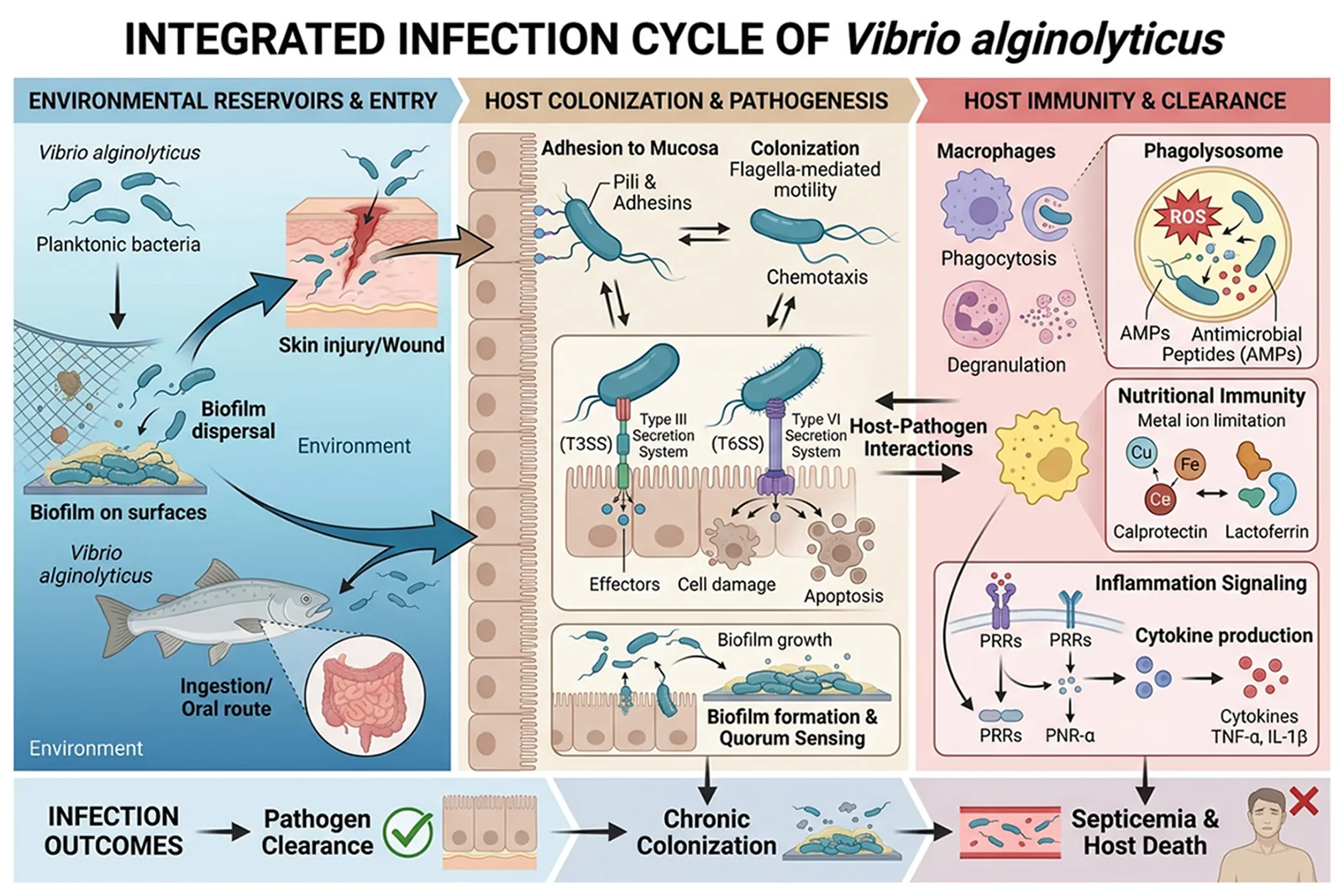
Infection cycle and host–pathogen interactions during *V. alginolyticus* pathogenesis. This schematic depicts the complete infectious lifecycle of *V. alginolyticus*, from environmental acquisition to host colonization and immune engagement. In the marine or aquaculture environment **(left)**, the bacterium exists as planktonic cells or within biofilms, and gains entry into the host through damaged skin, gill epithelia or ingestion of contaminated seafood. Following entry, *V. alginolyticus* adheres to mucosal surfaces (center) via flagella, pili and adhesins, and secretes T3SS/T6SS effector proteins that subvert host signaling, induce apoptosis or ferroptosis, and promote tissue damage. The pathogen subsequently establishes structured biofilm communities that enhance resistance to antimicrobials and immune clearance, while quorum-sensing circuits coordinate population-wide virulence gene expression. The host immune response **(right)** involves recruitment of macrophages and neutrophils, production of antimicrobial peptides and reactive oxygen species, induction of nutritional immunity (copper and iron sequestration), and activation of inflammatory signaling cascades (cytokine release). The dynamic interplay between bacterial virulence strategies and host defenses determines infection outcomes **(bottom)**: bacterial clearance, chronic colonization, or progression to septicaemia and mortality. Solid arrows indicate progression through the infection cycle, while bidirectional arrows denote reciprocal host–pathogen crosstalk.

In fish, the immune response to *V. alginolyticus* involves both humoral and cellular components. Glutathione peroxidase homologs from teleosts, such as PoGPx-7 (glutathione peroxidase homolog 7 from *Paralichthys olivaceus*), exhibit bactericidal activity by binding to *V. alginolyticus* via lipopolysaccharide and peptidoglycan, and activate host immune cells (72). The anti-lipopolysaccharide factor ALF4 has been identified as a key immune regulator against *V. alginolyticus* in the mud crab *Scylla paramamosain* (73).

Environmental stressors can modulate host susceptibility to *V. alginolyticus* infection. Low salinity stress does not significantly affect the proliferation or virulence of the bacterium but can significantly alter the microbial composition and immune response of infected oysters. The synergistic interaction between environmental stress and pathogen infection can cause mass mortalities by inducing host microflora imbalance and immune dysregulation (74). Understanding these environment–host–pathogen interactions is critical for predicting and managing disease outbreaks in aquaculture settings.

The metabolic interplay between host and pathogen represents another dimension of the host–pathogen interaction. The tricarboxylic acid (TCA) cycle plays a central role in this context. Genes encoding key TCA cycle enzymes, including phosphoenolpyruvate carboxylase (PckA), pyruvate dehydrogenase (PdhB), aconitate hydratase (AcnA), succinate dehydrogenase (SdhC), succinyl-CoA synthetase (SucC), and 2-oxoglutarate ferredoxin oxidoreductase (OorA), are downregulated under adhesion-reducing stress conditions (Cu^2+^, Pb^2+^, and low pH). Silencing of these genes reduces adhesion by 2.13- to 5.51-fold, with *acnA* showing the closest association with adhesion (75). Expression of TCA cycle genes is sensitive to pH and starvation, but not temperature, suggesting pathway-specific environmental responsiveness. The TCA pathway thus functions as an important metabolic sensor that links energy metabolism, precursor biosynthesis, and adhesion regulation (75).

Similarly, the oxidative phosphorylation pathway contributes to adhesion regulation. Six genes encoding subunits of cytochrome c oxidases, *coxA, coxB, coxC* (aa3-type) and *ccoN, ccoO, ccoQ* (cbb3-type), are downregulated under Cu^2+^, Pb^2+^, and low pH stress. Silencing of these genes reduces both cytochrome c oxidase activity and adhesion, with *ccoQ* showing the greatest impact on adhesion despite encoding a non-catalytic subunit (76). The expression of oxidative phosphorylation genes is sensitive to temperature, salinity, pH, and starvation, indicating that this pathway mediates adhesion regulation across diverse environmental conditions (76). These findings establish that central carbon and energy metabolism are integral components of the virulence regulatory network, not merely passive housekeeping functions.

### 7.1 Antimicrobial resistance

Antimicrobial resistance (AMR) in *V. alginolyticus* has emerged as a significant concern for both aquaculture and public health (77). The widespread use of antibiotics in aquaculture has contributed to the selection and dissemination of resistant strains (7).

### 7.2 Resistance prevalence

Surveillance studies have documented high rates of antibiotic resistance among *V. alginolyticus* isolates. A study of isolates from seafood and freshwater products in China found that 122 out of 128 isolates (95.31%) were resistant to at least one antibiotic category, and 2 isolates (1.56%) exhibited multidrug resistance (resistance to at least three antibiotic categories) (19). Similarly, *V. alginolyticus* isolates from aquaculture systems showed high resistance to ampicillin, erythromycin, cefotaxime, kanamycin, and doxycycline, with multiple antibiotic resistance (MAR) indices reaching 0.55–0.80 in isolates from the Hergla aquaculture farm (78). In Norwegian marine environments, resistance or reduced susceptibility to ampicillin was observed in 74% of *V. alginolyticus* isolates (79).

Seasonal surveillance in mariculture-impacted ecosystems reveals that the MAR index of environmental *Vibrio* isolates ranges from 0.01 to 0.03 across seasons, with three MAR strains detected (13). Notably, resistance to neomycin (6.78%) and intermediate resistance to neomycin (81.4%) and ciprofloxacin (31.07%) were observed, suggesting that these antibiotics may be under selective pressure and that resistance could emerge more broadly (13). The detection of MAR strains in spring, summer, and autumn indicates ongoing selection pressure in aquaculture environments.

### 7.3 Resistance mechanisms

Genomic analyses have identified five types of antibacterial drug resistance genes in *V. alginolyticus* (21). The most striking finding is the universal presence of β-lactam resistance genes: their prevalence is 100% among isolates, indicating a potential risk of natural resistance to β-lactam drugs. The *blaCARB* gene, encoding a carbenicillin-hydrolysing class A β-lactamase, is particularly prevalent, with detection rates of 98.04% in some studies (19, 21). Other commonly detected resistance genes include *tet* (encoding tetracycline resistance, 97.06%) and *fos* (encoding fosfomycin resistance, 4.90%) (21). Multidrug efflux pumps, such as the CRP-encoding efflux pump conferring resistance to fluoroquinolones and macrolides, have also been identified (80).

The discordance between genotypic and phenotypic resistance patterns has been noted; despite the ubiquitous presence of *blaCARB* genes in *V. alginolyticus*, both between- and within-species variations in ampicillin resistance are observed. This suggests that resistance gene carriage does not always translate to phenotypic resistance, possibly due to differences in gene expression or the presence of additional resistance mechanisms (81).

### 7.4 Implications

The high prevalence of AMR in *V. alginolyticus* has important implications. For aquaculture, antibiotic treatment options are becoming increasingly limited, necessitating the development of alternative control strategies. For public health, the potential for horizontal gene transfer of resistance determinants between *V. alginolyticus* and other *Vibrio* species, including human pathogens such as *V. cholerae* and *V. parahaemolyticus*, is a concern (80). The presence of mobile genetic elements in *V. alginolyticus* genomes facilitates such transfer (18). Virulence genes also show evidence of horizontal transfer; *tdh, trh*, and *hlyA* are detected in atypical host strains at rates of 6.32%, 15.52%, and 11.30%, respectively, indicating that virulence determinants move across species boundaries within the *Vibrio* genus (13). The antimicrobial resistance genes identified in *V. alginolyticus*, their prevalence rates, and their mechanisms of action are summarized in Table 2.

**Table 2:** Antimicrobial resistance genes and prevalence in *Vibrio alginolyticus*.

Resistance gene	Specific genes	Prevalence	Mechanism
β-Lactam resistance	*blaCARB* (class A β-lactamase)	98%−100% (19, 21)	Hydrolysis of β-lactam antibiotics
Tetracycline resistance	*tet* (efflux)	97.06% (21)	Tetracycline efflux
Fosfomycin resistance	*fos*	4.90% (21)	Fosfomycin inactivation
Multidrug efflux	CRP-encoding efflux pump	Variable (80)	Efflux of fluoroquinolones and macrolides
Chloramphenicol resistance	Type B chloramphenicol O-acetyltransferase	Detected (106)	Chloramphenicol acetylation
Oxytetracycline resistance	Phosphoribosyltransferase	Detected (106)	Oxytetracycline modification

*blaCARB*, gene encoding a CARB-type class A β-lactamase; *tet*, tetracycline resistance gene family; *fos*, fosfomycin resistance gene family; CRP, cyclic AMP receptor protein.

## 8 Detection and diagnosis

Accurate and rapid detection of *V. alginolyticus* is essential for both clinical diagnosis and surveillance in aquaculture and food safety contexts. Traditional culture-based methods, while reliable, are time-consuming and require expertise in species identification. Molecular methods have largely supplanted culture for many applications.

Several PCR-based approaches have been developed for *V. alginolyticus* detection. Real-time PCR (qPCR) targeting the *groEL* gene has been employed for detection and quantification (82). Multiplex real-time PCR assays using TaqMan probes have been developed for the simultaneous detection of multiple *Vibrio* species, including *V. alginolyticus* (83). A triple PCR method targeting the *blaCARB-17* gene of *V. parahaemolyticus* and the *gyrB* gene of *V. alginolyticus* enables simultaneous detection of *V. parahaemolyticus, V. alginolyticus*, and *V. cholerae* in aquatic foods (84). Digital PCR-based methods using microfluidic chambers offer quantitative detection with high sensitivity (85). To differentiate between viable and non-viable cells, propidium monoazide (PMA) has been coupled with multiplex real-time PCR (86). The limit of detection for some RT-PCR methods can be as low as 0.88 pg of DNA per reaction or 2 CFU per reaction (87).

## 9 Control strategies

The increasing prevalence of antimicrobial-resistant *V. alginolyticus* strains has spurred interest in alternative control strategies. These include phage therapy, vaccination, and probiotic approaches (88).

### 9.1 Phage therapy

Bacteriophages offer a promising alternative to antibiotics for controlling *V. alginolyticus* infections. Numerous lytic phages have been isolated and characterized. Phage R12Z, a broad-spectrum vibriophage, infects at least three *Vibrio* species, including seven strains of *V. alginolyticus*, and shows effective protection of shrimp postlarvae (89). Application of a *V. alginolyticus* phage to seabream resulted in a 93% decrease in the *Vibrio* population (90). Phage R23Z, a lytic siphovirus, significantly enhanced shrimp survival rates (>16%) during *V. alginolyticus* infection (91). The jumbo vibriophage pVa-22 possesses antivirulence activity and therapeutic potential (92). Importantly, phages can inhibit biofilm formation and eradicate mature biofilms (93).

However, phage therapy faces challenges, including the development of phage resistance. *V. alginolyticus* can acquire resistance through mutations in genes involved in the biosynthesis of phage receptors (94). The finding that QS positively regulates CPS-dependent phage infection suggests that inhibiting QS may inadvertently reduce phage therapy efficacy (63).

### 9.2 Vaccination

Vaccination represents another important control strategy for aquaculture. Several vaccine candidates have been developed against *V. alginolyticus* (88). Live-attenuated vaccines (LAVs) have shown particular promise. An attenuated live strain of the HY9901 mutant Δ*gr* provided protection against *V. alginolyticus* in pearl gentian grouper (95). Similarly, LAV candidates Δ*cpxA* and Δ*cpxR* have been evaluated (96). A *hop* (T3SS effector) mutant has been constructed and evaluated as a potential LAV in orange-spotted grouper (97). Subunit vaccines targeting flagellin proteins FlaD and FlaF, combined with the Montanide™ ISA 763B VG adjuvant, have induced protective immune responses in pearl gentian grouper (98). Autogenous vaccines, formulated from locally prevalent strains, can reduce antibiotic dependence (99). The Hfq mutant has also been proposed as a live attenuated vaccine candidate, given its role in stress adaptation and virulence (100).

### 9.3 Probiotics

Probiotics offer a sustainable and eco-friendly alternative to antibiotics for disease prevention in aquaculture, functioning through multiple mechanisms that collectively enhance host resistance while suppressing pathogen proliferation. The protective effects of probiotics are mediated through several complementary pathways: competitive exclusion of pathogens for adhesion sites and nutrients, secretion of antimicrobial compounds that directly inhibit Vibrio growth, interference with bacterial quorum sensing systems that coordinate virulence gene expression, and modulation of the host's innate immune responses (3). These mechanisms work synergistically to improve larval survival, enhance digestive enzyme activities, stabilize gut microbial communities, and maintain water quality in aquaculture systems (3, 101).

Among the most extensively studied probiotic genera in the context of *V. alginolyticus* control are *Bacillus, Lactobacillus*, and *Pseudoalteromonas*. *In vitro* antagonistic assays have demonstrated that *Lacticaseibacillus paracasei* and *Bacillus amyloliquefaciens* significantly inhibit the growth of *V. alginolyticus* and *V. vulnificus* in white shrimp (*Litopenaeus vannamei*) culture, with treated groups showing markedly lower Vibrio counts and ammonia levels compared to controls (101). *Lactobacillus plantarum* strains isolated from kefir have been shown to enhance non-specific immunity and immune-related gene expression in white shrimp; feeding with strain MRS18 at 10^9^ CFU/g for 14 days significantly improved survival rates following *V. alginolyticus* challenge (102).

Beyond *Bacillus* and *Lactobacillus*, other probiotic candidates have shown promise. *Pseudoalteromonas piscicida* strain SW-1, isolated from seawater in a clam farm, inhibits the growth of *V. alginolyticus in vitro* and improves clam survival following challenge. Mortality was only 11% when clams were simultaneously exposed to Pseudoalteromonas SW-1 and the pathogen, compared to 100% mortality in the pathogen-only group (103). Strains of *Bacillus licheniformis* YB-1 and *Bacillus megaterium* YB-2, isolated as potential probiotics for sea cucumber aquaculture, demonstrate strong antibacterial activity against *V. alginolyticus in vitro*, and the optimal combination achieved a 55% survival rate after pathogen challenge (104).

The integration of probiotics with modern aquaculture practices, such as biofloc technology and zero-water-exchange systems, has further strengthened their disease management potential by creating stable microbial ecosystems that naturally suppress Vibrio proliferation (101).

Despite these promising outcomes, the large-scale application of probiotics in aquaculture faces several practical challenges. A major limitation is stability, as maintaining microbial viability during feed formulation, storage, and exposure to variable environmental conditions such as temperature, salinity, and pH remains difficult (3). Many probiotic strains lose activity before reaching the host's gastrointestinal tract, thereby reducing their intended biological effects (3). Another challenge concerns applicability and consistency, as probiotic performance varies according to host species, developmental stage, culture conditions, and local microbial communities (3). Such strain-specific interactions necessitate customized formulations rather than universal “one-size-fits-all” solutions (3). Economic feasibility is also a key constraint, as continuous application is often required to maintain optimal probiotic levels, and production, formulation, and quality-control expenses can limit adoption, especially among small- and medium-scale farms (3). The lack of standardized regulatory frameworks and quality-assurance guidelines further complicates product evaluation and comparison across markets, leading to inconsistent performance of commercial formulations (3).

Nevertheless, the growing body of evidence supporting the efficacy of probiotics, combined with increasing pressure to reduce antibiotic use in aquaculture, underscores their potential as essential tools for sustainable disease management (101). Future research should focus on strain selection and optimization, formulation technologies that enhance stability and delivery, and the integration of probiotics into comprehensive health management strategies that combine biosecurity, environmental monitoring, and targeted interventions (3, 101).

## 10 Challenges and future research

Despite significant advances in understanding *V. alginolyticus*, several major challenges and priorities for future research remain. First, the molecular details of host–pathogen interactions, particularly the signaling pathways targeted by T3SS effectors and the mechanisms by which the bacterium evades host immune responses, warrant further investigation. The interplay between metabolic pathways (TCA cycle, oxidative phosphorylation) and virulence regulation represents a particularly promising area for future research (75, 76). Second, the ecological factors driving the emergence and dissemination of pathogenic and antibiotic-resistant strains are incompletely understood (17). The role of mobile genetic elements in the horizontal transfer of virulence and resistance genes merits closer scrutiny, particularly given the evidence of horizontal gene transfer of *tdh, trh*, and *hlyA* across *Vibrio* species (13). Third, the development of effective and practical control strategies for aquaculture settings requires continued research, particularly in the areas of phage therapy, vaccination, and probiotics (88). The complex interplay between QS, biofilm formation, and phage susceptibility highlights the need for integrated approaches that consider the multiple layers of regulation governing *V. alginolyticus* pathogenesis (63). Fourth, improved surveillance systems that integrate genomic, environmental, and clinical data are needed to detect emerging threats and inform public health interventions (5). The spatial and seasonal heterogeneity of *Vibrio* communities in mariculture-impacted ecosystems underscores the importance of targeted monitoring in high-risk areas (12, 13). Finally, the impact of climate change on the distribution and virulence of *V. alginolyticus*, and on the broader *Vibrio* community, represents a critical area for future investigation (5, 105). The emerging roles of ncRNAs and OMVs in virulence regulation (66–68, 70) open new avenues for therapeutic intervention, including RNA-based biopesticides and OMV-targeted strategies.

## 11 Conclusion

*Vibrio alginolyticus* has transitioned from a largely overlooked environmental organism to an emerging pathogen of significant concern in aquaculture and public health. Its success as a pathogen reflects a combination of factors: a versatile genome encoding diverse virulence determinants, sophisticated regulatory systems including quorum sensing, two-component systems, and non-coding RNAs, the capacity to form protective biofilms, the ability to deploy outer membrane vesicles for intercellular communication, and an expanding repertoire of antimicrobial resistance genes. The convergence of climate change, aquaculture intensification, and antimicrobial overuse has created conditions favorable for the continued emergence of *V. alginolyticus* as a threat to both aquatic animal health and human wellbeing. Addressing this threat will require an integrated, One Health approach that encompasses surveillance, basic research on pathogenesis, and the development of sustainable control strategies. Practical priorities include cross-sector sharing of surveillance data, improved aquaculture biosecurity and water-quality management, and stronger antimicrobial stewardship to reduce unnecessary antibiotic use and monitor resistance. These coordinated measures may support earlier outbreak detection and more sustainable control of *V. alginolyticus*.
